# Is It a “Hybrid Swarm”? Opportunities for More Informative Terminology in Conservation Genomics

**DOI:** 10.1002/ece3.74515

**Published:** 2026-10-24

**Authors:** Jillian N. Campbell, Elizabeth G. Mandeville, Nathan C. Lewis, Amanda V. Meuser

**Affiliations:** ^1^ Department of Integrative Biology University of Guelph Guelph Canada; ^2^ Department of Biodiversity Monitoring State Museum of Natural History Stuttgart Stuttgart Germany; ^3^ Department of Biology Northern Michigan University Marquette Michigan USA; ^4^ Department of Zoology University of British Columbia Vancouver Canada; ^5^ Department of Biology York University Toronto Canada; ^6^ Department of Zoology Stockholm University Stockholm Sweden; ^7^ Centre for Palaeogenetics Stockholm University Stockholm Sweden

**Keywords:** demographic swamping, genetic swamping, hybrid swarm, hybrid zone, hybridization

## Abstract

Hybridization is common in wild taxa, which may cause conservation concerns if hybridization occurs following disturbance or species introductions. High throughput DNA sequencing makes genomic analysis of many individuals feasible, vastly improving and promoting more studies on hybridization. However, more precise genomic identification of hybrids has not always extended to more precise use of terminology to describe hybrid ancestry. For hybridizing species of conservation concern, it is critical to understand hybridization dynamics precisely in order to effectively manage imperiled taxa. We conducted a review of 67 representative journal articles that use the term “hybrid swarm” in reference to hybridization, as variation in the usage of the term makes it difficult to understand which biological processes are being described. We found that “hybrid swarm” is used in several contexts across studies using various methods and numbers of genetic markers, reflecting vastly different statistical power and degrees of evidence for extensive hybridization. We encourage researchers working with genomic datasets to use clearer terminology when describing hybridization outcomes. We elaborate on scenario‐specific terminology to strengthen uniformity in hybridization language, which we hope will promote more precise descriptions of hybridization and improve the clarity of future research on hybridization.

## Graphical abstract

We conducted a comprehensive review of 67 journal articles that use the term “hybrid swarm” in reference to hybridization, as variation in the usage of the term makes it difficult to understand which biological processes are being described. We found that “hybrid swarm” is used in several contexts across studies using various methods and numbers of genetic markers, reflecting vastly different statistical power and degrees of evidence for extensive hybridization. We elaborate on scenario‐specific terminology to strengthen uniformity in hybridization language and encourage researchers working with modern genomic datasets to use clearer terminology when describing hybridization outcomes.

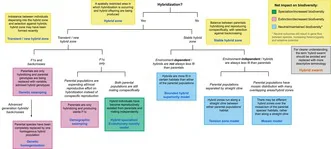

## 1 Introduction

Outcomes of hybridization between species can include speciation, introgression, and production of sterile hybrids (Allendorf et al. 2001). Hybridization has played a major role in the origins of diversity among species that we see today. For example, hundreds of new cichlid fishes resulted from natural hybridization between two divergent lineages of cichlids (Meier et al. 2017). However, hybridization can also threaten biodiversity when diverged species interbreed. Interbreeding species may experience demographic or genetic swamping, meaning populations of a species of interest are either physically or genetically replaced by hybrids (Rhymer and Simberloff 1996; Wolf et al. 2001; Todesco et al. 2016). For this reason, hybridization can be a conservation concern, particularly when hybridization occurs following a disturbance or species introduction (Allendorf et al. 2001). Research on at‐risk populations threatened by hybridization can help mitigate negative outcomes (Gese and Terletzky 2015, for example), but conservation success may be threatened by ambiguous classifications that obscure the true outcome of interspecific hybridization.

The term “hybrid swarm” was originally defined in 1926 as a dynamic group with hybrids that have intermediate traits that may be biased toward one parental species (Cockayne and Allan 1926). However, “hybrid swarm” appears to have been used in various contexts and with different meanings over the century since the term's origin. In many cases, “hybrid swarm” is used to describe scenarios where hybridization is perceived as undesirable, such as when a threatened native species hybridizes with a closely related introduced species. As genetic and genomic methods have improved, researchers have continued to refer to extensive hybridization as a “hybrid swarm”, even as high resolution genomic data has become more accessible for conservation‐focused studies (Narum et al. 2013). Using more genetic markers enables better identification of later‐generation hybrids and backcrosses, allowing the use of more specific terminology (McFarlane and Pemberton 2019). This is not merely an academic advancement, because increasingly precise genomic identification of hybrids can allow conservation practitioners to apply precisely targeted and ultimately more successful management strategies. In this review, we assembled a representative sample of empirical papers that focused on an ongoing “hybrid swarm” system, meaning that we excluded data for studies with systems that were descended from a hybrid swarm or could become a hybrid swarm in the future. We summarized how the term “hybrid swarm” has been used in the literature by investigating the species studied, methods used for hybrid identification (genetic versus phenotypic), generations of hybridization described, and number of species involved, which highlight the ambiguous use of the term “hybrid swarm”.

## 2 Methods

### 2.1 Data Collection

To determine how “hybrid swarm” has been used in a representative sample of the scientific literature, we collected articles using a standardized search in R. Using four packages, easyPubMed (Fantini 2019, version 2.13), remotes (Csárdi et al. 2021, version 2.4.0), litsearchr (Grames et al. 2019, version 1.0.0), and plyr (Wickham 2011, version 1.8.6), we created a custom script that searched and pulled publications from the PubMed database. Search terms “hybrid swarm” and “biology” were used to provide bounds for the PubMed database search. After the initial search, our results contained many articles that were not pertinent to the scope of this review. Many of these off‐target articles contained the word “particle” in addition to “hybrid swarm” and “biology”. Therefore, we modified our query terms to not include the word “particle”. In each of our two searches (conducted on February 8, 2021 and March 27, 2023), we pulled 74 articles, following the default batch size in the example we worked from. The search provided key information such as the title, publishing journal, author list, and date of publication, which were then used to find and download the full articles.

Each article was read independently by two different readers and assessed for fit in the scope of this review. A total of 6 different readers were involved in reading and assessing all articles. We classified each paper as either relevant or not to hybridization in an evolutionary biology context and either describing an ongoing hybrid swarm or not. Articles were given a “no” in the “ongoing hybrid swarm” category for one of two reasons: either the authors stated that the organisms in their study system were descended from a hybrid swarm or that their system could become a hybrid swarm in the future, rather than actively existing as one during the time of study. For each paper deemed relevant, two different researchers read and manually collected the following data: type of evidence for hybridization (genetic, phenotypic, etc.), taxonomic group (e.g., bird, fish, plant), genus, number of species involved in hybridization, number of individuals sampled, number of genetic loci employed (or NA if no genetic data were used), number of sampling locations, years of hybridization, genetic marker type (or NA if no genetic data were used), number of generations of hybrids (F1, F2, or beyond), number of generations of backcrossing, and finally, binary responses for whether anthropogenic disturbances, non‐anthropogenic disturbances, or species introductions were present in the system and whether data were available for the study. If the data collected by the two readers for a single article did not match, any differences were manually evaluated and reconciled by an additional reader.

### 2.2 Data Analysis

We created a timeline of some of the major events relating to the use of “hybrid swarm” and other major events related to genetics (Figure 1). We also plotted summary statistics on the cleaned data (Figure 2) and created a Sankey plot depicting outcomes of hybridization for all relevant papers (Figure 3). Finally, we created a decision tree to represent proper hybridization terminology under different scenarios (Figure 4). Figures 1 and 4 were created using Google Slides. Figures 2 and 3 were created using R Statistical Software (R Core Team 2021, version 4.1.2), specifically using the tidyverse (Wickham et al. 2019, version 1.3.1), scales (Wickham et al. 2022, version 1.2.1), patchwork (Pedersen 2023, version 1.1.3), and ggsankey (Sjoberg 2021) packages.

**FIGURE 1:**
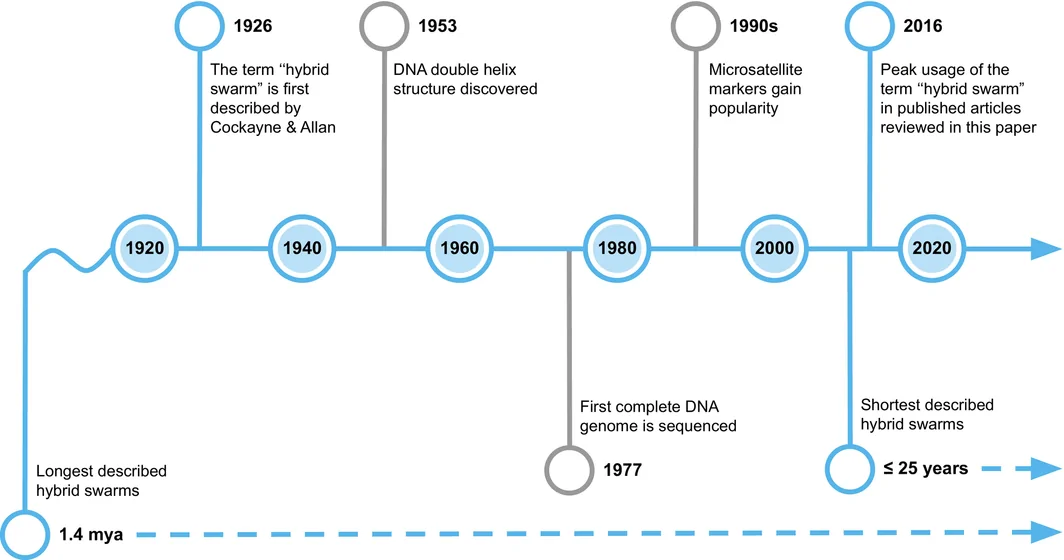
Timeline depicting usage of the term “hybrid swarm” (blue) and important historic events (gray) surrounding genomic research. Figure adapted from Martínez‐Pérez et al. (2022). mya, million years ago.

**FIGURE 2:**
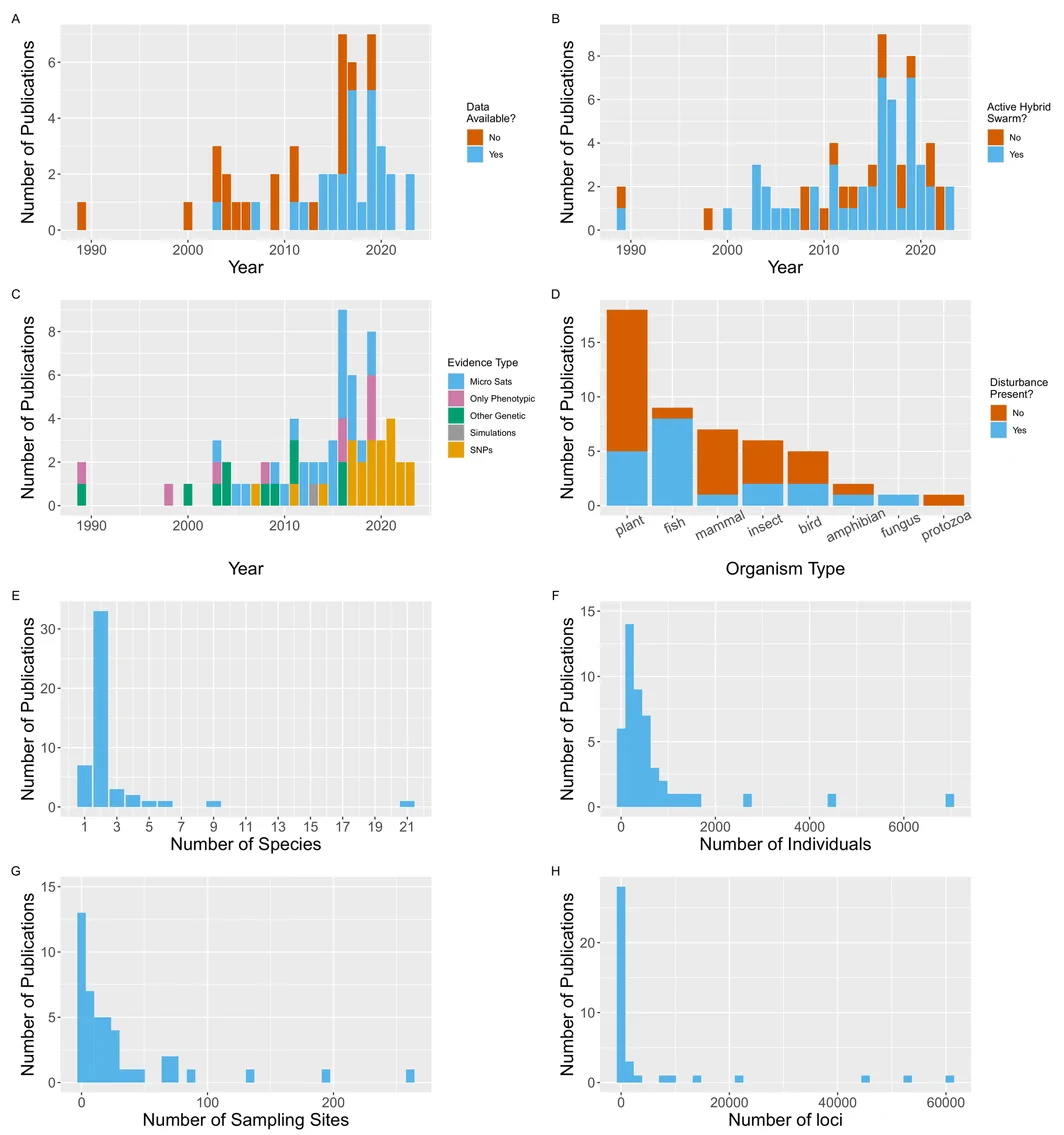
Summary statistics on the term “hybrid swarm”, in the 49 papers which described hybrid swarms (unless otherwise specified). (A) Number of publications which have published data available, by year. (B) Number of publications per year, labeled by active hybrid swarm status (for all 67 relevant papers). (C) Number of publications per year, labeled by evidence type (for all 67 relevant papers). (D) Number of publications with disturbance present, sorted by type of organism. (E) Number of publications per number of species studied. (E) Number of publications per number of individuals studied. (F) Number of publications per number of sampling sites studied. (G) Number of publications per number of sampling sites studied. (H) Number of publications per number of loci studied (one outlier with 299,305 loci was excluded for clarity).

**FIGURE 3:**
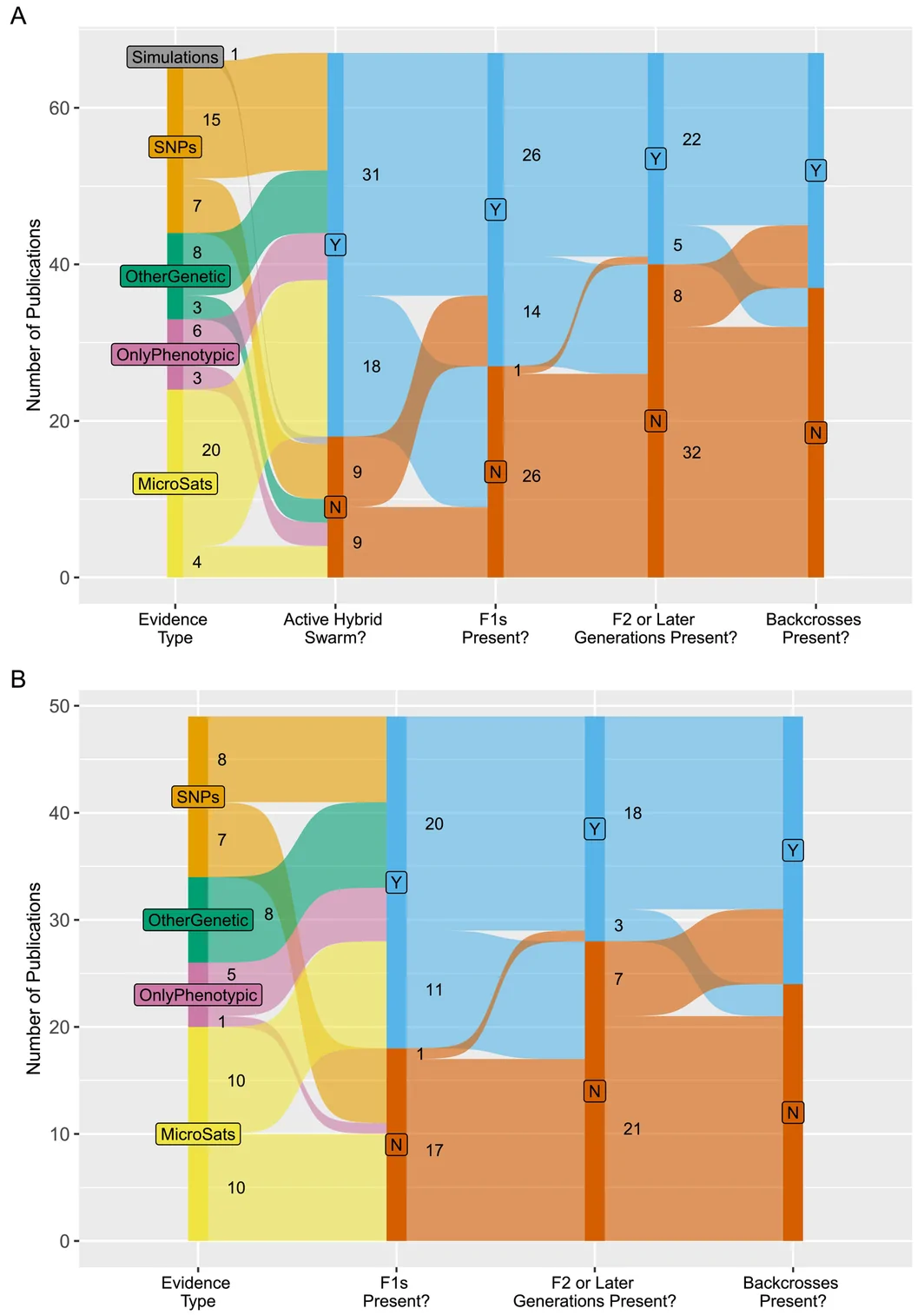
Sankey plot depicting hybrid outcomes. Evidence type describes the type of evidence that a paper used to detect hybrids in a system; papers that used a type of genetic marker may have also used non‐genetic evidence as well. (A) All 67 relevant papers. (B) Only the 49 papers depicting active hybrid swarms.

**FIGURE 4:**
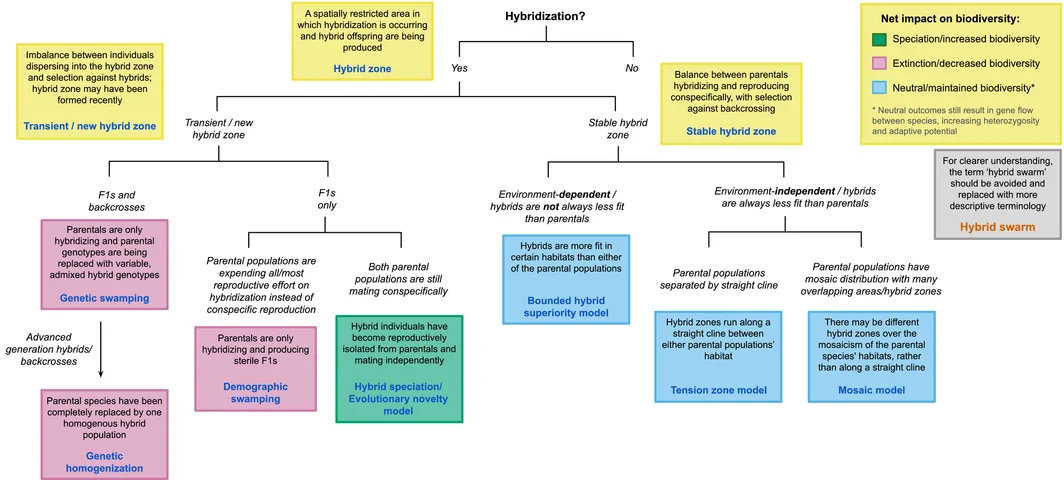
Decision tree depicting terminology to describe the potential evolutionary outcome of hybrid zones. The term “hybrid swarm” has been used as a catch‐all term to describe many different cases of hybrid zone and should therefore be replaced with more specific terminology instead.

We used Fleiss' kappa to assess agreement among readers for paper relevance and number of hybrid generations. An analysis of paper relevance was also done adjusting for blanks in the raw data. If a reader left the relevance column blank but proceeded to fill out other information about the paper, these empty slots were then assumed to indicate that the reader found the paper relevant. When assessing agreement for number of hybrid generations, only papers where at least one reader deemed the paper as relevant were included. Papers that were categorized as not relevant by both readers were removed.

## 3 Results

From a total of 148 papers between the two data pulls, there was an overlap of 37 papers leaving 111 unique papers. From these, we classified 67 as relevant to hybridization in the context of evolutionary biology and 49 as including active hybrid swarms. The first usage of “hybrid swarm” within the papers pulled by our search was in 1989 (Figure 2A–C). The search algorithm prioritized more recent studies, so this is not an exhaustive list of papers using the term “hybrid swarm”, but this focus on the past 35 years of publications is a representative sample of the literature which suits our goal of assessing current usage of “hybrid swarm” in the field. Within the papers we surveyed, there was a peak in usage of “hybrid swarm” in the recent past (within the last 10 years) as compared to > 10 years ago (Figures 1 and 2A–C).

With how we grouped study organisms, plants were the most common group to be referred to as a hybrid swarm and amphibians, fungi, and protozoa were the least common (Figure 2D). Two‐species hybrid systems were the most commonly studied, followed by single‐species systems, due to the many papers on hybridization between sub‐species (Figure 2E). There was substantial variation in the number of individuals per study, though most included 500 or fewer individuals (Figure 2F). A similar trend was observed with the number of sampling sites and loci used in a study, where some outliers used many, but most studies used very few sampling sites or loci (fewer than 25 or 100, respectively) (Figure 2G,H).

Single nucleotide polymorphisms (SNPs) were more frequently used to describe hybrid swarms in recent years, while micro‐satellite marker usage is becoming less frequent (Figure 2C). Use of both genetic evidence and phenotypic evidence has been fairly stable over the years, with genetic evidence being used more overall (Figure 2C). Data from publications describing hybrid swarms were published more frequently in recent years (Figure 2A). Disturbances are usually mentioned by studies that described fish systems as hybrid swarms, while the opposite is true for studies on plants (Figure 2D).

Most of the active hybrid swarm systems (31/49) had F1s present (Figure 3). Two common outcomes were either having all 3 hybrid types present (F1s, F2 or later generations, and backcrosses) or having none (18/49 and 17/49, respectively). About half of all papers (25/49) recorded having backcrosses present. A small number (4/49) of papers had F1s present but no other hybrids, and only one system had F2s or later generation hybrids without also having F1s.

Using Fleiss' kappa, paper relevance was found to have moderate agreement among readers (κ=0.532). However, when adjusting for blanks in the raw data where relevance is assumed based on continued paper analysis, paper relevance showed almost perfect agreement (κ=0.872). Conversely, number of hybrid generations had only slight agreement (κ=0.163).

## 4 Discussion

The first use of the term “hybrid swarm” was by Cockayne and Allan (1926). They stated that a hybrid swarm is dynamic, unlike a species, which is static, and that in a hybrid swarm there are hybrids that look more or less intermediate, often biased toward one parental species. However, this definition is quite broad, and our literature review and data analysis suggest that use of the term does not always fit a consistent definition. The papers we reviewed described many very different and distinct evolutionary scenarios as hybrid swarms. Many of the papers we reviewed stated that the presence of a large number of backcrosses or exclusively backcrosses in a hybrid zone are characteristics of a hybrid swarm (Anttila et al. 2000; Glotzbecker et al. 2016; Haines et al. 2019; Jacquemyn et al. 2016; Keim et al. 1989; Lavretsky et al. 2019; Opiro et al. 2017; Stemshorn et al. 2011; Tihon et al. 2017). However, we found much evidence to the contrary in our analysis as only about half of the articles investigating active hybrid swarms described the presence of backcrosses (Figure 3) and four described first generation hybrids but no later generation hybrids or backcrosses (Campoy et al. 2019; Mallet et al. 1998; Marczewski et al. 2016; Van Der Sluijs et al. 2008). More articles suggested that both backcrossing and mating between hybrids are found in hybrid swarms than just backcrossing alone (Glotzbecker et al. 2016).

Seventeen papers were classified as having hybrids present but not having discerned between hybrid types, indicating that they potentially either did not have suitable data to discern between hybrid types or it was not a consideration of the study (Anttila et al. 2000; Taylor et al. 2006; Mende et al. 2016, as examples). However, modern studies on hybridization that use SNP data from many loci have more power to accurately describe hybridization outcomes (McFarlane and Pemberton 2019), potentially enabling classification of hybrids and eliminating the need for a vague, catch‐all term like “hybrid swarm”. Many of the papers that we reviewed used the term “hybrid swarm” when they were unable to confidently differentiate hybrid generations or backcrosses from one another. Thus, while an initial impression from the literature may be that a hybrid swarm can be clearly defined as the presence of many backcrosses in a hybrid zone, we have found that not to be the case. From our data, we found that most of these hybrid swarms had F1s present, some had continued hybrid generations, and about half had backcrosses present (Figure 3); overall, there was no clear consensus as to what hybrid outcomes are present in a hybrid swarm.

For the representative sample of papers we reviewed, we found that use of “hybrid swarm” peaked between 2016 and 2019 (Figure 2A,B,D). We attribute this increase in usage to the likely increase in studies published on hybridization in wild systems using genomic data, as the cost of sequencing many individuals has decreased over the last two decades and the use of SNP data has increased while microsatellite markers decreased (Davey et al. 2011; Lou et al. 2021; Narum et al. 2013) (Figure 2C). We emphasize the importance of using precise language to avoid further confusion, specifically as genomic datasets improve the discernment of hybrid outcomes within a hybrid zone.

The papers we reviewed revealed no consensus on how stability or duration may define a hybrid swarm. While stability and duration are not typically considered with usage of the term, there are examples where these factors have been used as important criteria for designation of hybridizing species as a hybrid swarm. For example, while describing hybridization between two amphibian species amid an anthropogenic disturbance, Fitzpatrick and Shaffer (2007) used both “hybrid zone” and “hybrid swarm”, and implied that hybrid zones are areas of stable hybridization while hybrid swarms are expanding and threatening to cause genetic and/or demographic swamping of the species. However, we found that “hybrid swarm” was also used to describe systems with and without disturbances (Figure 2D). Additionally, the term was used to describe hybrid zones that have existed for vastly different time periods, including some present for 25 years or less at the time of publication (Anttila et al. 2000; Erickson et al. 2020; Glotzbecker et al. 2016; Kirk et al. 2005; Knutson et al. 2019; Rangel et al. 2016; Ward et al. 2012) as well as hybrid zones which have potentially existed for thousands of years (Li et al. 2016; McDevitt et al. 2009) or over one million years (Dupuis and Sperling 2015) (Figure 1). This inconsistency is somewhat understandable given that historical details of hybridization are not always known, especially in understudied systems. Indeed, few studies of hybridization have been conducted over long timescales, with consistent genetic methods throughout (except for a few notable studies; e.g., Muhlfeld et al. 2017). Incorporating temporal data would improve our understanding of hybridization outcomes and may aid in conservation efforts. For example, if it is known that a hybrid zone has existed for decades to millennia and both parental populations still remain distinct, this system could be described as a stable hybrid zone and may not necessarily be as great a conservation concern as another instance of hybridization where one or both parental species are at risk of extinction (Dupuis and Sperling 2015).

Furthermore, the confusion surrounding the term “hybrid swarm” can be seen by agreement between trained readers of the literature used in this review, as quantified by Fleiss' kappa. Readers had almost perfect agreement on papers that were relevant to the study (κ=0.872). This showed that readers were able to distinguish studies using the term easily. However, readers were only in slight agreement over the number of hybrid generations (κ=0.163). In many instances readers would leave a blank or “unclear” under this category. This further establishes the difficulty of interpreting the hybridization dynamics present in a study when non‐specific language is used.

Based on our synthesis of published papers, we argue that many “hybrid swarms” are simply instances of hybrid zones and that the term “hybrid swarm” is imprecise and may be misleading. Instead, we advocate for more precise descriptions of hybridization dynamics in a system, as enabled by genomic data (Figure 4). Crystal et al. (2016) stated that hybrid swarms are “characterized by high phenotypic variability among individuals with extremely diverse genotypes.” This is corroborated by Dejaco et al. (2016) who also stated that “when two distantly related genomes fuse, hybrid swarms consisting of lineages with different admixture proportions can emerge… hybrid swarms can act as cradle of novel, highly adapted genotypes”. Many studies have shown that hybridization can cause novel phenotypes, unique from that of either parental species (e.g., Hegarty et al. 2008; Rieseberg et al. 1999; Stelkens and Seehausen 2009). While describing evolutionary novelty as resulting from a hybrid swarm is not problematic in a conservation context, we suggest that this catch‐all term of “hybrid swarm” may still not be the best way to describe this phenomenon. Perhaps something along the lines of “cradle of adaptive novelty” may be more suitable, such as used in Dejaco et al. (2016) or the analogies of hybrid zones being “windows” or “laboratories” for studying evolution, as used by Harrison (1990).

To describe evolutionary outcomes or conservation implications of hybridization, it would be more appropriate to use another, preexisting term to describe the hybrid zone, as shown in Figure 4. Demographic swamping and genetic swamping are two such more precise terms, used to describe new or transient hybrid zones (Todesco et al. 2016). Demographic swamping occurs when the waste of reproductive efforts between two species on the production of sterile F1 hybrids causes one species to go extinct or be extirpated, while genetic swamping is continual mating between two species and their fertile offspring, causing the replacement of all parental species individuals with hybrids or backcrosses. Allendorf et al. (2022) stated that hybrid swarms are “populations in which all individuals are hybrids by varying numbers of generations of back‐crossing with parental types and mating among hybrids” but later go on to say that “this phenomenon [hybrid swarms] has generally been referred to as genetic swamping” (Chapter 13, pages 273 and 286 of Allendorf et al. 2022), showing a precedent for using “genetic swamping” in place of “hybrid swarm”. Some papers do explicitly distinguish these outcomes. For example, Pinto et al. (2005) stated that “the degree of mixing between hybridizing forms may range from formation of a hybrid swarm to genetic assimilation of one form by the other.” This definition seemingly distinguishes the formation of a hybrid swarm from genetic swamping, or potentially from genetic homogenization. However, if a hybrid zone can be maintained without an overall loss of biodiversity via demographic or genetic swamping, this could be considered a stable hybrid zone (Todesco et al. 2016). Since these two types of swamping via hybridization may require different conservation interventions, we suggest that more precise language would be beneficial in many of the studies we reviewed.

A large body of theoretical and empirical work describes different types of stable hybrid zones. Stable hybrid zones may result from a balance between dispersal into the hybrid zone and selection against hybridization (Harrison 1986). This is the premise of the Tension Zone model, where hybrids are less fit than parentals, independent of their environment, maintaining the balance between hybridization and selection (Barton and Hewitt 1985). The Mosaic model is similarly environment‐independent when it comes to lower hybrid fitness, but there may be several distinct hybrid zones over the mosaicism of the parental species' habitats, rather than along a straight cline such as in the Tension Zone model (Harrison 1986; Arnold 1997). Finally, under the Bounded Hybrid Superiority model, hybrids may be more fit in certain habitats than either parental species, though this is environment‐dependent (Moore 1977). If the balance between dispersal and selection breaks down, stable hybrid zones can become transient and outcomes can often result in species extinction/decreased biodiversity. Likewise, recently formed hybrid zones often lack this balance and the stability of new hybrid zones may be unclear. Human disturbance has been often shown to form new hybrid zones which may result in hybridization‐led extinction (Allendorf et al. 2001; Todesco et al. 2016). The Bounded Hybrid Superiority model can contribute to hybrid speciation if (a) the hybrids are able to reproduce independently, producing F2, F3, etc. generations of hybrids, and (b) if the parental populations also stop producing hybrids (Abbott et al. 2013). If only (a) is satisfied, the stable hybrid zone remains. However, the Bounded Hybrid Superiority model differs slightly from the Evolutionary Novelty model, as under the latter, hybrids may also be more fit than one or both parental species in the latter's habitat (Arnold 1997). Overall, these descriptions of stable hybrid zones suggest that we have long had the terminology to better describe hybrid zones, and argue against use of “hybrid swarm” to refer to various hybrid types.

As a demonstration of the power of using more precise language in our analysis of populations of conservation concern, we offer an example from a system that we have worked on extensively, *Catostomus* fishes. Following the introduction of a non‐native species into the range of two closely related fish species, hybridization became widespread. Initial efforts to assess the situation with genetic data (specifically AFLPs; Amplified Fragment Length Polymorphisms) revealed extensive hybridization (Douglas and Douglas 2008; McDonald et al. 2008), but lacked the resolution and geographic sampling to fully describe the outcomes of hybridization. However, the use of the terms “hybrid swarm” and “muttsucker” to describe the hybrids created substantial alarm surrounding conservation policy, leading to extensive effort to manage populations of the native species. When sequenced using newer technologies and described more precisely, hybrids were revealed to be mostly first‐generation and symmetrical advanced‐generation backcrossed hybrids (Mandeville et al. 2017), including some individuals with ancestry from all three species, but there was extensive geographic variation in outcomes of hybridization. Additionally, higher resolution genomic data revealed that the two species native to the study area had historically hybridized prior to the recent introduction (Bangs et al. 2018). Describing the hybrids more precisely suggested new avenues for potential management, including protecting certain tributaries important for spawning. We assessed outcomes of a conservation intervention and found that while the method might be promising in years with optimal hydrological conditions, the interannual variation in hybridization outcomes and interannual variation in high flow river conditions might make this approach less feasible (Campbell 2023). By moving away from the use of the term “hybrid swarm” and carefully quantifying hybridization outcomes, we were able to better serve conservation goals and more clearly assess options for management.

### 4.1 Recommendations

We argue that the terms “hybrid zone” and “hybridization”, along with detailed genomic analysis of hybridization, can be used to replace “hybrid swarm” in most, if not all cases. We observed these terms used interchangeably in many studies (e.g., Fitzpatrick and Shaffer 2007; McDevitt et al. 2009). We recommend that authors clearly describe the types of hybrids found in their study system, including the abundance of F1s, backcrosses, or continued generations of hybrids (F2s, F3s, etc.). With the abundance of high‐resolution genomic data being produced, precisely describing hybrid types is becoming easier (McFarlane and Pemberton 2019) and substantial data is now available for even non‐model organisms (as predicted by Narum et al. 2013). We do recognize that not all systems will benefit from this recommendation. Instances where genomic data does not meet the requirements or hybridization outcomes are more complex (e.g., multi‐species hybrids or ghost introgression) would constitute situations where description may be more convoluted. In these cases, we still argue against the use of the term “hybrid swarm”, as the negative connotation associated with this term may also affect interpretation of the research. Instead, we recommend using a more neutral term such as “hybrid zone” with a thorough and clear explanation of the dynamics of these systems. This allows flexibility for researchers with unclear data but also avoids the use of the ambiguous “hybrid swarm”.

The types of hybrids in a hybrid zone at one time are not necessarily indicative of the potential evolutionary outcome of this hybrid zone. As noted in Abbott et al. 2013, “the pattern of contemporary hybridization is potentially only a single snapshot of a complex and continuously changing interaction”. However, for hybrid zones that have been studied over more than one time point, researchers may also speculate upon these potential evolutionary outcomes. We recommend referring to the decision tree (Figure 4) when describing specific hybrid zone scenarios. Here, we define a “hybrid zone” as a spatially restricted area in which hybridization is occurring and hybrid offspring are being produced (Galbusera et al. 2025). We have drawn on the literature to compile existing descriptions of hybrid zones and classified them as either describing stable hybrid zones or transient/new hybrid zones (Arnold 1997; Barton and Hewitt 1985; Harrison 1986; Moore 1977; Todesco et al. 2016).

Methods for studying hybridization have changed extremely rapidly over the last few decades. The use of ancient or historical DNA data is now increasing in prevalence and making it possible to study evolution over the last million years (Dalén et al. 2023). However, when looking back over these evolutionary timescales, describing hybrid zones becomes even more difficult. For example, Columbian mammoths were shown to have been a hybrid species formed from at least two hybridization events between resident North American mammoths and early waves of woolly mammoth migration into North America (van der Valk et al. 2021). After the first hybridization event around 500 thousand years ago, it is likely that neither parental species remained in North America—all or a very large proportion of mammoth individuals after that point are assumed to be the Columbian hybrids (van der Valk et al. 2021; Lister and Dalén 2024). Was this a genetic homogenization event? Or could this have fit the Evolutionary Novelty model with later regional extinction of the parental populations? We have very few fossils from these distant time points and even less genomic data, plus  uncertainty surrounding the geographical location of the hybrid zone (Lister and Dalén 2024), thus, it can be tempting to apply broad terms such as “hybrid swarm”. But even when faced with sparse data, it is best to use a neutral term such as “hybrid zone” or “hybridization complex” with potential suggestions to the type of hybridization outcomes, if possible.

This review was prompted by the large variety of terminology used to describe hybridization and disconnect with current datasets. We hope that our findings will provoke thoughtful discussion and consideration of the terminology best suited to describe hybridization in an era when genomic data provides unprecedented opportunity to better define outcomes of hybridization. In particular, we believe that conservation and management programs for species where hybridization is a relevant concern will benefit from better, more precise use of terminology when discussing outcomes of hybridization.

## Author Contributions

**Jillian N. Campbell:** conceptualization (equal), data curation (lead), formal analysis (equal), investigation (equal), methodology (equal), project administration (equal), validation (equal), visualization (equal), writing – original draft (equal), writing – review and editing (equal). **Elizabeth G. Mandeville:** conceptualization (equal), funding acquisition (lead), project administration (supporting), supervision (lead), writing – original draft (equal), writing – review and editing (equal). **Nathan C. Lewis:** formal analysis (equal), investigation (equal), visualization (supporting), writing – review and editing (equal). **Amanda V. Meuser:** conceptualization (supporting), formal analysis (equal), investigation (equal), methodology (equal), project administration (equal), visualization (equal), writing – original draft (equal), writing – review and editing (equal).

## Funding

This work was supported by the National Sciences and Engineering Research Council Undergraduate Student Research Award, Colorado Parks and Wildlife Species Conservation Trust Fund (SCA20BXYZ), and the Canada First Research Excellence Fund.

## Conflicts of Interest

The authors declare no conflicts of interest.

## Data Availability

R scripts and raw data files used for the PubMed search and creating figures can be found on GitHub (https://github.com/amanda‐meuser/Hybrid_Swarm_Paper/) and on the Dryad data repository (https://doi.org/10.5061/dryad.w3r228189).
